# Optimally invasive mitral valve surgery: a safe and effective approach

**DOI:** 10.1186/1749-8090-8-S1-O269

**Published:** 2013-09-11

**Authors:** A Amiri, E Delmo Walter, R Hetzer

**Affiliations:** 1 Cardiothoracic Surgery, Deutsches Herzzentrum Berlin, Berlin, Germany

## Background

We aim to report our data on the efficacy and safety of using an optimally invasive sternum-sparing approach to MV surgery.

## Methods

Between April 2007 and December 2012, we used a sternum-sparing but optimally invasive approach to MV surgery on 99 patients (mean age 60.71±12.9 years) with mean preoperative EF and LVEDD of 53.8±11.4 % and 56.13±6.9 mm, respectively. Twenty seven patients had previous coronary artery bypass and MV surgeries. All patients had severe mitral insufficiency (MI) from chordal rupture; prolapse of the anterior leaflet, paravalvular leak, endocarditis, floppy MV, and from previous MV surgery. The optimally invasive approach was a right-sided anterolateral thoracotomy at the 5th intercostal space with an approximately 10 cm skin incision. Cardiopulmonary bypass (CPB) was through either cannulation of the ascending aorta or femoral artery with direct bicaval cannulation. Modified Gerbode-Hetzer plication for ruptured chordae and modified Paneth-Hetzer posterior annulus shortening annuloplasty, for annulus dilatation or leaflet prolapse were employed. Paravalvular leaks were closed. Intraoperative TEE was used to evaluate the adequacy of repair or replacement.

## Results

The mean CPB and cross-clamp time were 134.4±52.2 and 56.63±29.7 minutes, respectively. All patients were discharged with either absence or minimal MI. Mean postoperative EF improved to 65.13±8.7% while mean postoperative LVEDD decreased to 51.6±7.0 mm. Sixty-seven percent of patients were extubated within 24 hours, 86% required minimal postoperative analgesia and all had satisfactory functional results on follow-up (mean 4.2±1.07 years) aside from good cosmesis. Freedom of reoperation is 100% until the last follow-up.

## Conclusions

This new innovative approach is a safe and effective option to MV surgery, reduces surgical trauma, increases patients’ functional capacity and satisfaction, no morbidity, with 100% freedom from reoperation in 4 years.

